# Mechanisms of Mitotic Kinase Regulation: A Structural Perspective

**DOI:** 10.3389/fcell.2018.00006

**Published:** 2018-02-05

**Authors:** Julie P. I. Welburn, A. Arockia Jeyaprakash

**Affiliations:** Wellcome Trust Centre for Cell Biology, School of Biological Sciences, University of Edinburgh, Scotland, United Kingdom

**Keywords:** mitotic kinase, mitosis, phosphorylation, structure, mechanism, substrate

## Abstract

Protein kinases are major regulators of mitosis, with over 30% of the mitotic proteome phosphorylated on serines, threonines and tyrosines. The human genome encodes for 518 kinases that have a structurally conserved catalytic domain and includes about a dozen of cell division specific ones. Yet each kinase has unique structural features that allow their distinct substrate recognition and modes of regulation. These unique regulatory features determine their accurate spatio-temporal activation critical for correct progression through mitosis and are exploited for therapeutic purposes. In this review, we will discuss the principles of mitotic kinase activation and the structural determinants that underlie functional specificity.

## Introduction

Protein phosphorylation is a key regulatory mechanism influencing various cellular processes such as cell growth, cell motility, cell differentiation and cell division. Most notably, protein phosphorylation peaks during mitosis and the timing coincides with the cell division-related chromosomal and cytoskeletal reorganization (Dephoure et al., 2008; Olsen et al., 2010). Consequently, mitotic protein kinases are considered central players orchestrating the mitotic progression and accurate spatio-temporal regulation of their activity becomes essential for error-free chromosome segregation. Many mitotic kinases are well characterized in terms of their structure and function. The list includes but is not limited to CDK (cyclin-dependent kinase; CDK1 and CDK2), Aurora (Aurora-A and Aurora-B) and Plk (Polo-like kinase; Plk1, Plk) families, Bub1, Haspin and Mps1 (see also a focused review on NIMA family of kinase by Fry et al, in this issue). Though mitotic kinases share significant structural similarities, their cellular localization, enzymatic activity and substrate specificity are determined by diverse mechanisms.

In this review, we will summarize our understanding of how structurally conserved and distinct features determine correct spatio-temporal regulation of kinase activity during mitosis.

## Generally conserved structural features of a kinase catalytic domain

**Figure 1:**
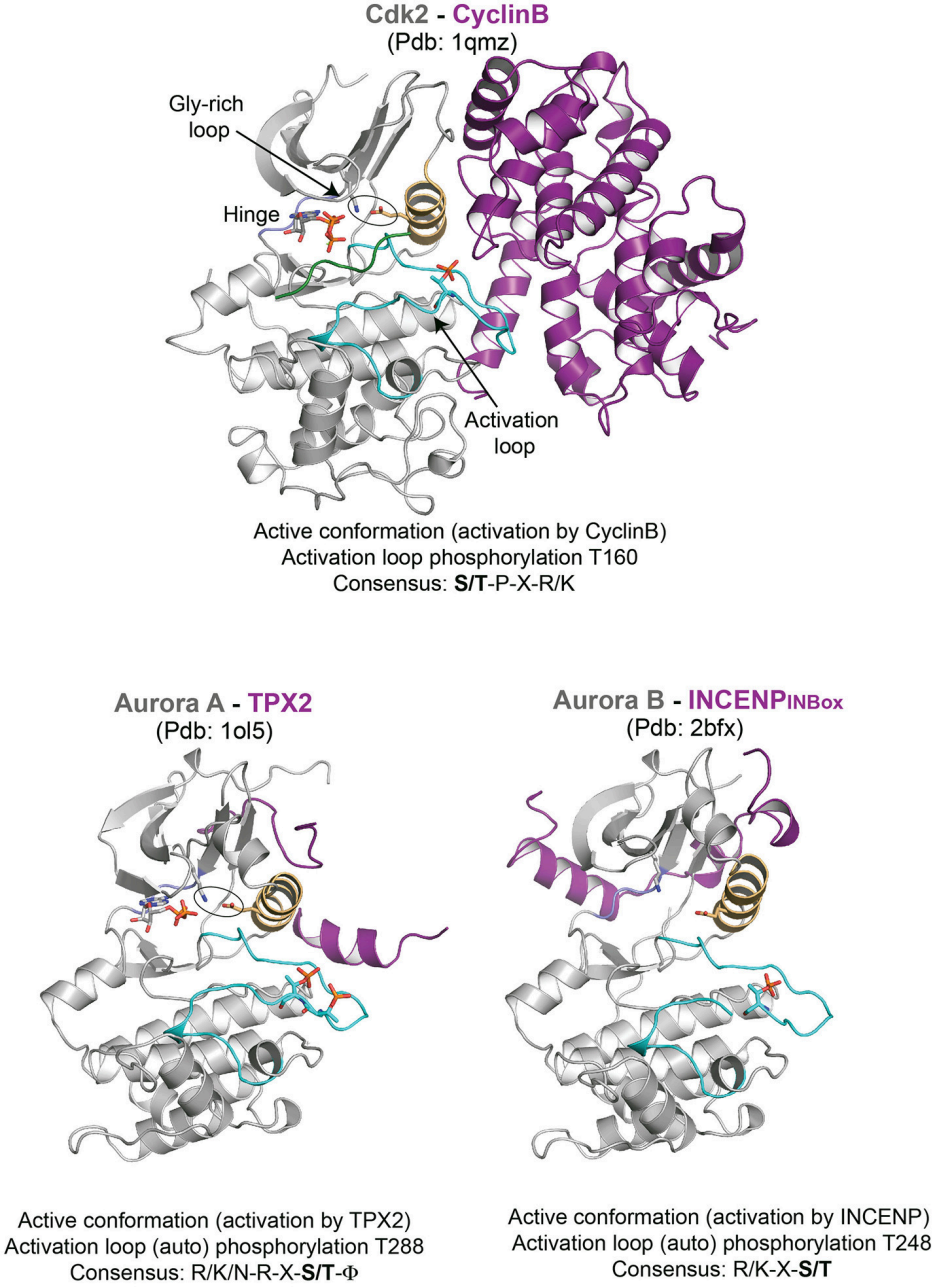
Representative structures of mitotic kinases I. Key structural features are highlighted: C-helix (light orange), hinge region involved in ATP binding (blue), and activation segment (cyan). The characteristic structural feature of an active enzyme, the salt bridge between a Lys and Glu from the N-terminal lobe and the C-helix is highlighted in stick representation.

Most protein kinases have a bilobal catalytic domain of ≈290 amino acids with an N-terminal lobe made of a β-sheet and one α-helix (known as the C-helix) and an α-helical C-terminal lobe (reviewed in Johnson et al., 1996; Bayliss et al., 2012). The active site that transfers the γ-phosphate of ATP to the substrate is buried at the interface between the two lobes (Figure 1). When the kinase is catalytically active, the C-helix packs against the N-terminal lobe (Kobayashi et al., 1992; Brown et al., 1995; Johnson et al., 1996; Bayliss et al., 2003; Sessa et al., 2005; Petri et al., 2007; Endicott et al., 2012). The ATP binding pocket is largely conserved across kinases and is surrounded by relatively less conserved pockets often exploited for inhibitor design (Noble et al., 2004). The peptide backbone of the hinge region connecting the N- and C-terminal lobes makes hydrogen bonds to the adenine ring of the ATP, while nonpolar aliphatic residues lining the pocket interact with the purine structure. A glutamate from the C-helix and a lysine from the N-terminal lobe make a salt bridge (a conserved feature of an active kinase achieved by the packing of the C-helix against the N-terminal lobe) and stabilize the α- and β-phosphate groups of ATP. Note that when generating a kinase dead mutant, this lysine usually is targeted. The glycine-rich loop in the N-terminal lobe also stabilizes the ATP β- and γ-phosphate groups, in addition to its important regulatory role in controlling the access of the substrate to the kinase active site. Finally two magnesium ions in the ATP binding pocket coordinate the phosphate groups to ensure transfer of the γ-phosphate to the hydroxyl acceptor group (Johnson et al., 1996; Bayliss et al., 2012; Endicott et al., 2012).

The C-terminal lobe contains an activation segment that varies in length and sequence but is defined by the well-conserved amino acid motifs *DFG* and *APE* (D-[L/Y]-G and [S/P]-P-E are uncommon variations) at each end (Bayliss et al., 2012; Endicott et al., 2012). This activation segment is usually remodeled during kinase activation through phosphorylation, although intra- or inter-molecular protein binding can also trigger activation segment remodeling (Bayliss et al., 2012; Endicott et al., 2012). When non-phosphorylated, the activation segment is disordered and auto-inhibits the kinase by obstructing the substrate binding site. Phosphorylation of the activation segment on a phosphoacceptor serine or threonine typically activates the kinase by stabilizing the substrate-binding site. The negatively charged phosphate group is engaged by the basic residues contributed by the C-helix, the N-terminal lobe and the activation segment to stabilize the substrate-binding platform and hence the active conformation of the kinase (Endicott et al., 2012).

## Mechanisms of kinase activation

### Activation by inter or intra molecular protein interaction

Mechanism of mitotic kinase activation was first established for the cyclin-dependent kinase (CDK) family (Evans et al., 1983; Felix et al., 1990; Kobayashi et al., 1992). Pioneering studies in yeast, sea urchin and Xenopus showed that phosphorylation and cyclins controlled the activity of CDKs to drive the cell cycle. Cyclin levels are regulated throughout cell cycle both at the transcriptional and proteolytic level, which in turn activates CDK activity temporally (Evans et al., 1983; Pines and Hunter, 1989; Felix et al., 1990). Cyclins have a cyclin box domain that binds to the N-terminal lobe and the C-helix of the kinase domain (Kobayashi et al., 1992; Brown et al., 1995; Petri et al., 2007). This triggers the packing of the C-helix to remodel the ATP binding site (Brown et al., 1999a, 2015). This mode of activation is broadly conserved in CDK1 (Cdc2 and Cdc28 in budding and fission yeast respectively), which upon cyclin B binding initiates mitosis (Santamaria et al., 2007; Gavet and Pines, 2010; Diril et al., 2012).

The Aurora family of kinases mainly Aurora A and Aurora B, are among the well characterized protein kinases (Bayliss et al., 2003; Sessa et al., 2005; Zorba et al., 2014). These exhibit basal level of kinase activity on their own, but their full activation requires specific binding partners: Aurora A, implicated in centrosome maturation and mitotic spindle stability, involves TPX2 while Aurora B activity essential for destabilizing erroneous kinetochore-microtubule attachment and cytokinesis requires INCENP (which together with Borealin and Survivin functions as the *C*hromosomal *P*assenger *C*omplex, CPC). Though the activation loop auto-phosphorylation is sufficient for basal activity, facilitating the right conformation of the loop is required for full activation, and is achieved by the binding of TPX2 and INCENP to Aurora A and Aurora B kinases, respectively (Bayliss et al., 2003; Sessa et al., 2005). A short N-terminal fragment of TPX2 binds between the N-lobe and activation loop of Aurora A and by doing so it stabilizes the activation loop in a fully active conformation. This makes the activation loop phosphorylation (pT288) inaccessible for counteracting phosphatases and thus maintains the fully active state of Aurora A. In the case of Aurora B, the C-terminal INBOX domain of INCENP wraps around the Aurora B N-lobe interacting with the C-helix, however is not sufficient for the full activation of Aurora B (at least on the basis of the relative orientation of C-helix with respect to the N-terminal lobe as seen in the crystal structures) (Figure 1). It has been postulated that additional interactions between the phosphorylated TSS motif downstream of INBOX with Aurora B might be required for full activation (Sessa et al., 2005).

**Figure 2:**
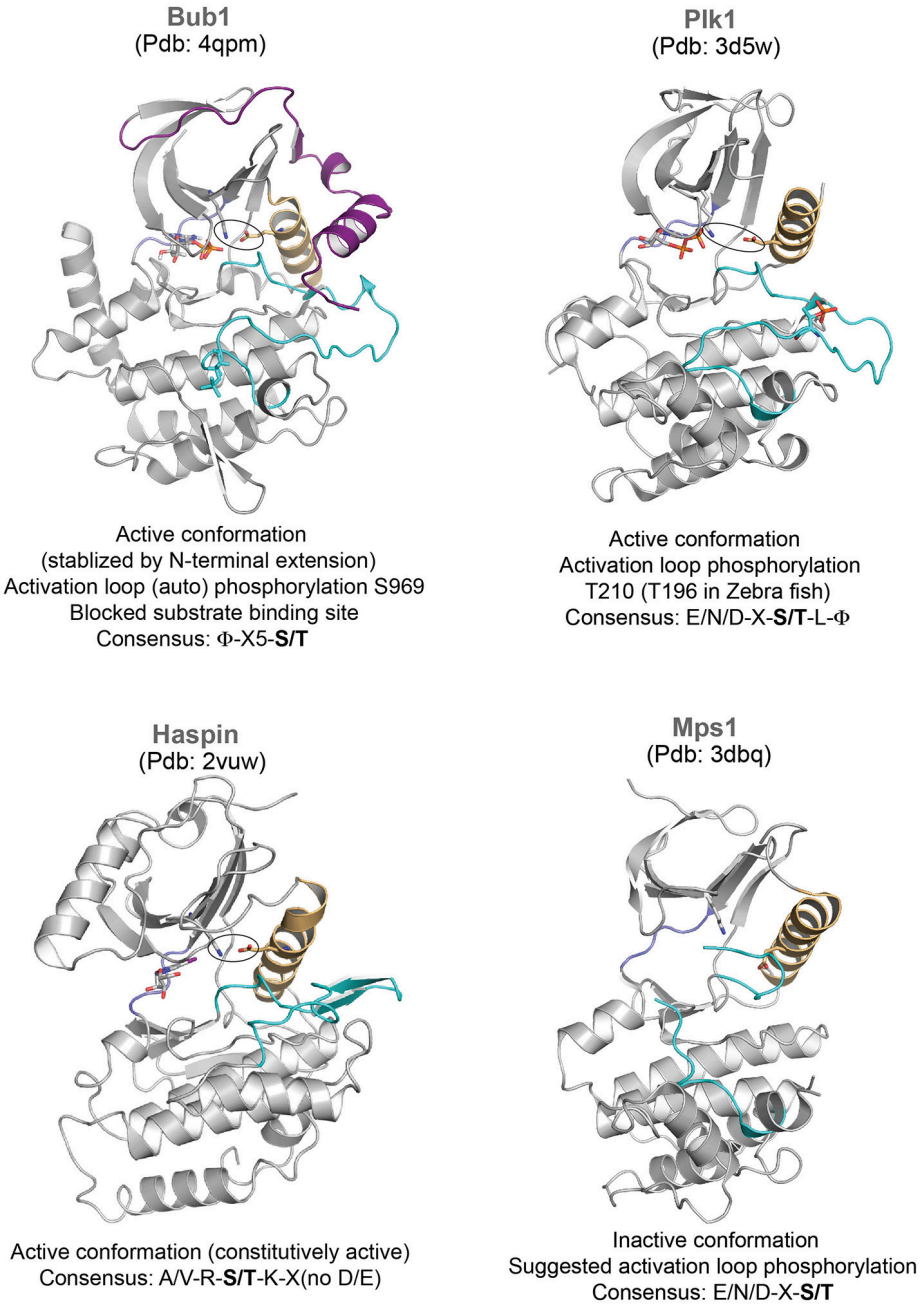
Representative structures of mitotic kinases II. Key structural features are highlighted: C-helix (light orange), hinge region involved in ATP binding (blue), and activation segment (cyan). The characteristic structural feature of an active enzyme, the salt bridge between a Lys and Glu from the N-terminal lobe and the C-helix is highlighted in stick representation.

Bub1 is a kinetochore-associated protein kinase which regulates the Spindle Assembly Checkpoint (SAC) and kinetochore-microtubule attachment (Elowe, 2011). Cdc20, Mad1/2 and Histone H2A appear as major substrates, while Bub1 also plays a scaffolding role at kinetochores to recruit the pseudokinase BubR1 (Tang et al., 2004; Yamagishi et al., 2010; Suijkerbuijk et al., 2012; Overlack et al., 2015, 2017; Jia et al., 2016). Structural characterization of Bub1 kinase domain reveals the role of its N-terminal extension in positioning the C-helix and stabilizing the conformation of the activation segment (Lin et al., 2014) (Figure 2). However, the activation segment conformation seen in the crystal structure does not appear to be suitable for substrate binding and activation loop phosphorylation has been implicated to enhance kinase activity and substrate recognition (Lin et al., 2014) (Figure 2).

### Regulation by phosphorylation

Activation segment phosphorylation regulates kinase activity of several kinases during mitosis. Both Aurora A and Aurora B kinases undergo auto-phosphorylation within their activation segments (T288 and T248 in Aurora A and Aurora B, respectively) which is essential for their increased kinase activity (Bayliss et al., 2003; Sessa et al., 2005). Bub1 undergoes constitutive autophosphorylation in its activation loop throughout cell cycle, which is suggested to trigger reorganization in the substrate binding site and enhance kinase activity (Lin et al., 2014). Likewise, Mps1 auto-phosphorylates multiple residues in its activation loop to activate itself (Mattison et al., 2007; Wang et al., 2009). The crystal structure of Mps1 kinase dead mutant revealed that it adopts an inactive conformation (Figure 2), where the activation loop is disordered and displaces the C-helix (Chu et al., 2008; Wang et al., 2009). Auto-phosphorylation of the activation loop may reorient and allow correct positioning of the C-helix for full activity. The activation segment phosphorylations of CDK1 and Plk1 are mediated by other kinases—CAK (*CDK* Activating *K*inase belonging to the family of CDKs) (Russo et al., 1996; Brown et al., 1999b), Aurora family of kinases (Aurora A in mammals, Jang et al., 2002; Macurek et al., 2008; Seki et al., 2008 and Aurora B in Drosophila, Carmena et al., 2012a; Xu et al., 2013; Kachaner et al., 2014), respectively.

Phosphorylation within the kinase module has also been used to inactivate kinases. For example, the wee1 kinase inhibits CDK1 by phosphorylating the Tyrosine 15 in the N-terminal glycine-rich loop and obstructing access of the substrate to the kinase active site (Russell and Nurse, 1987; Parker and Piwnica-Worms, 1992; McGowan and Russell, 1993; Welburn et al., 2007). This inhibition is reversed by the phosphatase Cdc25 (Frazer and Young, 2012).

### Kinases with constitutive activity

Unlike most mitotic kinases, the haspin kinase responsible for the histone H3 Thr3 phosphorylation (essential for the centromere localization of Aurora B/CPC) and the budding yeast Hrr25 (Casein kinase) critical for meiosis co-orientation of kinetochore do not require the activation loop phosphorylation and their kinase domains are constitutively active (Eswaran et al., 2009; Villa et al., 2009; Ye et al., 2016). However, the intrinsic activity of the haspin kinase is inhibited during prophase by its conserved basic N terminus (haspin 380-399, human numbering) preceding the kinase domain. During prophase to metaphase transition, the concerted activities of CDK1 and Plk1 relieve the auto-inhibition through phosphorylation in the N-terminal segment, and thus restrict the haspin activity to metaphase (Ghenoiu et al., 2013).

## Mechanisms for achieving substrate specificity

Kinases achieve substrate specificity through multiple mechanisms in mitosis (Ubersax and Ferrell, 2007; Johnson, 2011): by specific recognition of amino acid sequence flanking the phosphorylation site or/and by employing a domain capable of recognizing substrates marked by priming phosphorylation. In addition, specific subcellular localization of kinases also facilitates substrate specificity by spatially restricting the kinases.

### Consensus phosphorylation motifs

Kinase substrate specificity is generally determined by the architecture of the substrate binding site, which might select negatively against certain residues flanking the phosphorylation site. The identification of substrates *in vitro* and *in vivo* thus far has helped define kinase substrate specificity. Typically about 4-6 amino acids flanking the phospho-acceptor residue P can contribute to the selectivity of kinases for their substrate. The molecular basis for substrate recognition mainly comes from the structural work on CDK2/cyclin A bound to its substrate (Brown et al., 1999a). This work identified S/T-P-X-R/K as a preferred consensus phosphorylation motif for CDK. The activation segment adopts a conformation such that the carbonyl group of V164 is unusually strained and cannot form a hydrogen bond with the backbone of P+1 residue. As a result, other residues other than a proline at this position are unfavored. The preference for K/R at P+3 arises from an interaction between the phosphorylated and highly negatively charged threonine in the activation loop of CDK and the positively charged side chain of K/R (Heald and McKeon, 1990; Peter et al., 1990; Ward and Kirschner, 1990; Brown et al., 1999a).

The identification of multiple budding yeast kinetochore Aurora B kinase substrates, allowed to define an Aurora consensus phosphorylation sequences (Cheeseman et al., 2002) and subsequent identification of other Aurora substrates (Andrews et al., 2004; Lan et al., 2004; Cheeseman et al., 2006; DeLuca et al., 2006). Aurora kinases preferentially phosphorylate substrates with basic residues at P-3 and P-2 and containing a hydrophobic residue at P+1. Plk1 has a preference for an acidic residue at P-2 and a hydrophobic residue at P+1 (Toyoshima-Morimoto et al., 2001; Nakajima et al., 2003). An integrated approach combining biochemical, proteomic and structural biology methods identified substrates of the Haspin kinase, its consensus substrate recognition motif and their mode of interaction (Maiolica et al., 2014). Crystal structure analysis revealed that residues P-2 (A), P-1 (R), P (S/T) and P+1 (K) were specifically recognized within a deep pocket and thus providing a rational for the proposed consensus recognition motif A/V-R-S/T-K (Maiolica et al., 2014). In the case of Bub1, *in vitro* phosphorylation of kinetochore proteins by Bub1 followed by phosphorylation-directed staining and mass spectrometric analyses identified many prospective Bub1 substrates with a putative consensus motif ϕ-X_5_-S/T (Breit et al., 2015).

The use of peptide libraries against kinases has greatly contributed to the identification of optimal peptide sequence motifs and “anti-motifs” for kinases (Hutti et al., 2004). For example, Plk1, Aurora A and Aurora B strongly discriminate against proline at P+1 (Alexander et al., 2011). However, kinases do tolerate variations in the consensus sequence of their substrates. Proteomic studies combined with known phosphorylation consensus sites and bioinformatics represent powerful ways to uncover and validate new substrates *in vivo* (Dephoure et al., 2008; Mok et al., 2010; Kettenbach et al., 2011; Santamaria et al., 2011). Quantitative proteomics also inform substrate specificity of kinases not previously known. For example, such studies revealed that Mps1 and Plk1 share the same substrate preference (Dou et al., 2011; Petrone et al., 2016). Overall the substrate consensus motif plays a determining role in kinase-substrate interactions.

### Substrate priming

Kinases may use docking sites or sites that are primed by another kinase to enhance their substrate selectivity. For example, to bind and to be phosphorylated by Plk1, a substrate generally needs to be primed by another kinase (Lee et al., 2008). Plk family members have two Polo-box domains (PBD), that recognize Plk1 substrates (typically containing S-S/Tp-P) primed by other kinases such as Cdk1. By doing so the PBD of Plk1 recruits the catalytic domain to the phosphorylated substrates (Cheng et al., 2003; Elia et al., 2003a,b; Barr et al., 2004). Known mitotic substrates of Plk1 are the checkpoint proteins Bub1, BubR1 and Wee1 (Watanabe et al., 2005; Qi et al., 2006; Elowe et al., 2007). There have also been evidence for Plk1 “self-priming” (reviewed in Lee et al., 2008).

CDK-cyclins also use docking sites to recognize and phosphorylate temporally their substrates. Certain cyclin partners have a hydrophobic docking patch that recognizes an “RXL” motif on substrates 40Å away from the catalytic site of the CDK active site (Schulman et al., 1998; Brown et al., 1999a). A subset of substrates have a “RXL” motif recognized by a cyclin and are phosphorylated earlier in the cell cycle because they have a higher affinity for the CDK-cyclin complex (Loog and Morgan, 2005; Koivomagi et al., 2011). Substrates that do not have this docking site are phosphorylated later during mitosis or may not be recognized by the CDK-cyclin complex (Koivomagi et al., 2011). Overall the docking site interactions increase the local concentration of the substrate and ensure accurate spatio-temporal substrate phosphorylation essential for correct mitotic progression (Brown et al., 2015).

### Subcellular localization

Many mitotic kinases rely on spatial targeting to phosphorylate their specific substrates. This restricts the activity of the kinase to generate gradients of kinase activity. The most well characterized spatially-targeted kinases are Aurora A and B kinases and they appear to share the same substrate specificity (Fu et al., 2009). However Aurora A predominantly associates with centrosomes and mitotic spindle, while Aurora B is localized at centromeres and kinetochores. Centrosome association of Aurora A is mediated by TPX2, whereas Aurora B (and hence the CPC) localization to centromeres is mediated by phosphorylation marks on Histone H3 (Thr3) and Histone H2A (Thr120) created by Haspin and Bub1, respectively (reviewed in Carmena et al., 2012b; Kitagawa and Lee, 2015). While the Survivin subunit of the CPC recognizes the histone H3 mark, Sgo1 recognizes the H2A mark (Kelly et al., 2010; Wang et al., 2010; Yamagishi et al., 2010). The CPC indirectly recognizes histone H2A via the interaction of CDK1-phosphorylated Borealin with histone H2A bound Sgo1 (Tsukahara et al., 2010). Due to their distinct subcellular localization, while Aurora B phosphorylates substrates such as histone H3, kinetochore proteins and spindle midzone proteins (Gruneberg et al., 2004; Guse et al., 2005; Nunes Bastos et al., 2013), Aurora A phosphorylates a number of centrosomal and spindle-localized substrates (Sardon et al., 2010).

The molecular basis for the recruitment of Mps1 kinase to the outer kinetochore is also well established. Multiple kinases including CDK1, Aurora B, Plk1, and Mps1 itself are implicated in Mps1 kinetochore targeting (Morin et al., 2012; Nijenhuis et al., 2013; von Schubert et al., 2015). In addition, direct interaction of Mps1 with the Ndc80 complex is crucial for its localization and function (Hiruma et al., 2015; Ji et al., 2015). While the N-terminal extension of Mps1 directly interacts with the CH-domain of Ndc80 (adjacent to the microtubule binding region), the conserved middle region of Mps1 interacts with the Nuf2 CH domain. The affinity of Mps1 is higher for Aurora B-phosphorylated Ndc80, indicating that Aurora B promotes Mps1-Ndc80 interaction in response to unattached kinetochores (Ji et al., 2015). Mps1 can then phosphorylate Knl1 to activate the spindle checkpoint.

## Summary

High resolution mechanistic understanding of kinase regulation is essential not only to define how kinases achieve error-free cell division, but also to exploit the differences in their regulatory mechanisms to specifically target them in mitosis-related human disorders. Structural studies of kinases thus far have provided key insights into the similarities and differences in the modes of activation and regulation of many mitotic kinases. Although kinases possess a broadly conserved catalytic core, their level of kinase activity and substrate specificity are determined by specific inter/intra molecular interactions and phosphorylation. In this review, we summarize how key structural regulatory elements such as the relative orientation of the C-helix, activation segment conformation and spatial regulatory elements responsible for correct kinase sub-cellular localization achieve accurate kinase function. However, there are still many open questions, particularly on factors determining the graded level of kinase activation and its implications on their mitotic role. More structural analyses of kinases in complex with their regulatory binding partners with and without bound substrates, and their functional implications in cells will undoubtedly further advance our mechanistic understanding of this essential class of mitotic regulators.

## Author contributions

JW and AJ have made a substantial, direct and intellectual contribution to the work, and approved it for publication.

### Conflict of interest statement

The authors declare that the research was conducted in the absence of any commercial or financial relationships that could be construed as a potential conflict of interest.
